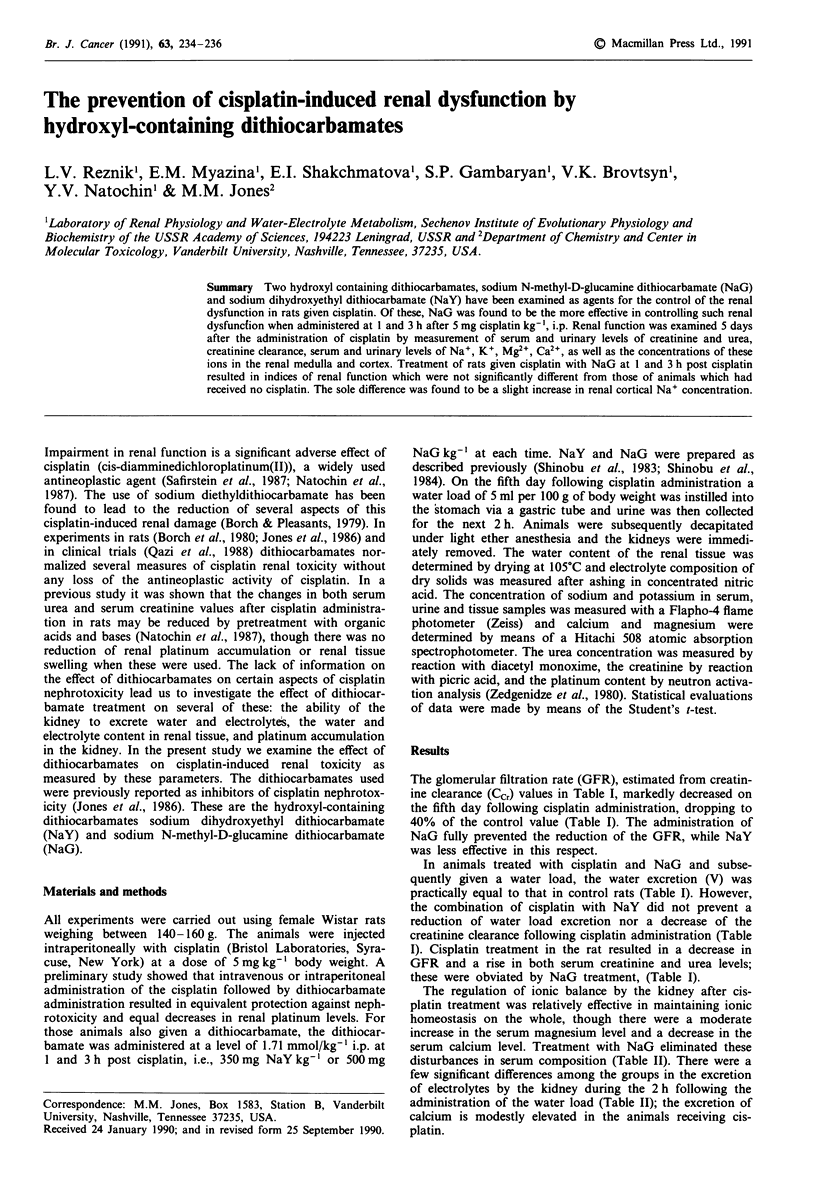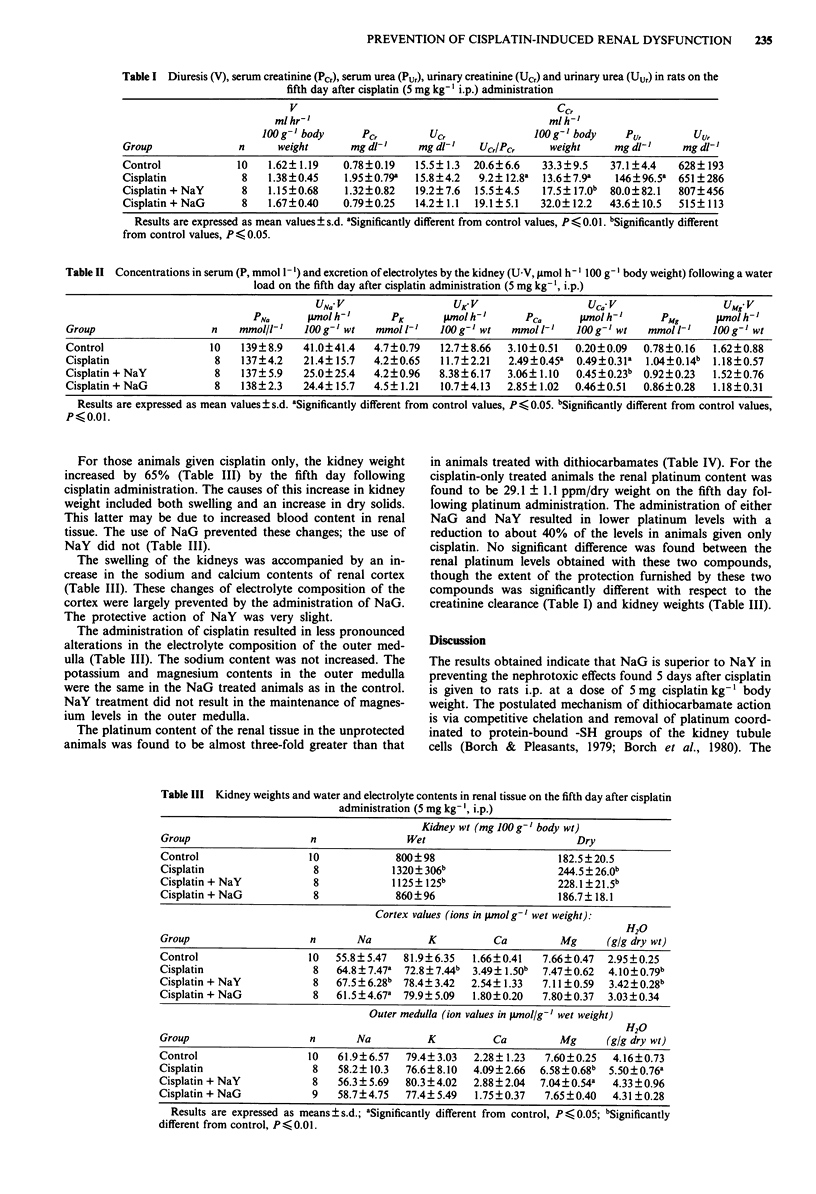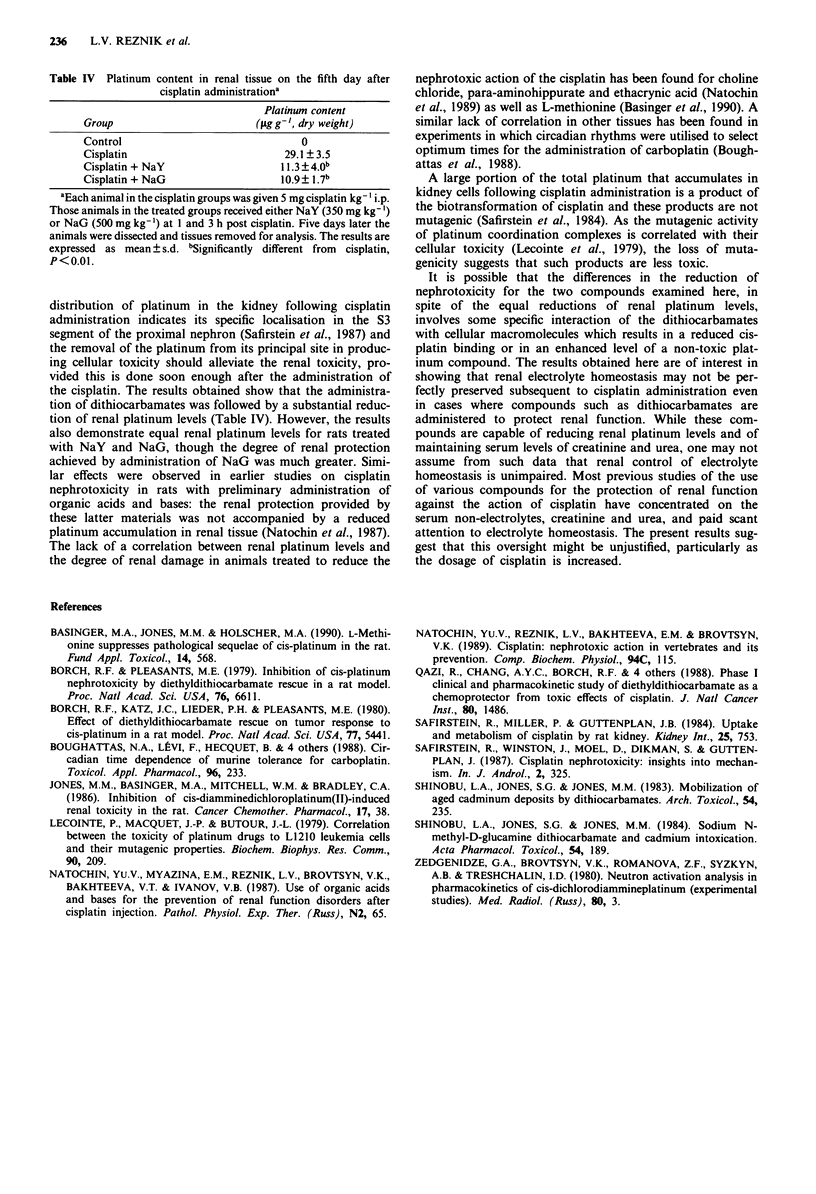# The prevention of cisplatin-induced renal dysfunction by hydroxyl-containing dithiocarbamates.

**DOI:** 10.1038/bjc.1991.55

**Published:** 1991-02

**Authors:** L. V. Reznik, E. M. Myazina, E. I. Shakchmatova, S. P. Gambaryan, V. K. Brovtsyn, Y. V. Natochin, M. M. Jones

**Affiliations:** Laboratory of Renal Physiology and Water-Electrolyte Metabolism, Sechenov Institute of Evolutionary Physiology and Biochemistry, USSR Academy of Sciences, Leningrad.

## Abstract

Two hydroxyl containing dithiocarbamates, sodium N-methyl-D-glucamine dithiocarbamate (NaG) and sodium dihydroxyethyl dithiocarbamate (NaY) have been examined as agents for the control of the renal dysfunction in rats given cisplatin. Of these, NaG was found to be the more effective in controlling such renal dysfunction when administered at 1 and 3 h after 5 mg cisplatin kg-1, i.p. Renal function was examined 5 days after the administration of cisplatin by measurement of serum and urinary levels of creatinine and urea, creatinine clearance, serum and urinary levels of Na+, K+, Mg2+, Ca2+, as well as the concentrations of these ions in the renal medulla and cortex. Treatment of rats given cisplatin with NaG at 1 and 3 h post cisplatin resulted in indices of renal function which were not significantly different from those of animals which had received no cisplatin. The sole difference was found to be a slight increase in renal cortical Na+ concentration.


Br. J. Cancer (1991), 63, 234-236 © Macmillan Press Ltd., 1991

### The prevention of cisplatin-induced renal dysfunction by hydroxyl-containing dithiocarbamates

L.V. Reznik[1], E.M. Myazina[1], E.I. Shakchmatova[1], S.P. Gambaryan[1], V.K. Brovtsyn[1], Y.V. Natochin[1] & M.M. Jones[2]

[1]*Laboratory of Renal Physiology and Water-Electrolyte Metabolism, Sechenov Institute of Evolutionary Physiology and Biochemistry of the USSR Academy of Sciences, 194223 Leningrad, USSR and* [2]*Department of Chemistry and Center in Molecular Toxicology, Vanderbilt University, Nashville, Tennessee, 37235, USA.*

**Summary** Two hydroxyl containing dithiocarbamates, sodium N-methyl-D-glucamine dithiocarbamate (NaG) and sodium dihydroxyethyl dithiocarbamate (NaY) have been examined as agents for the control of the renal dysfunction in rats given cisplatin. Of these, NaG was found to be the more effective in controlling such renal dysfunction when administered at 1 and 3 h after 5 mg cisplatin $kg^{-1}$, i.p. Renal function was examined 5 days after the administration of cisplatin by measurement of serum and urinary levels of creatinine and urea, creatinine clearance, serum and urinary levels of $Na^+$, $K^+$, $Mg^{2+}$, $Ca^{2+}$, as well as the concentrations of these ions in the renal medulla and cortex. Treatment of rats given cisplatin with NaG at 1 and 3 h post cisplatin resulted in indices of renal function which were not significantly different from those of animals which had received no cisplatin. The sole difference was found to be a slight increase in renal cortical $Na^+$ concentration.

Impairment in renal function is a significant adverse effect of cisplatin (cis-diamminedichloroplatinum(II)), a widely used antineoplastic agent (Safirstein *et al.*, 1987; Natochin *et al.*, 1987). The use of sodium diethyldithiocarbamate has been found to lead to the reduction of several aspects of this cisplatin-induced renal damage (Borch & Pleasants, 1979). In experiments in rats (Borch *et al.*, 1980; Jones *et al.*, 1986) and in clinical trials (Qazi *et al.*, 1988) dithiocarbamates normalized several measures of cisplatin renal toxicity without any loss of the antineoplastic activity of cisplatin. In a previous study it was shown that the changes in both serum urea and serum creatinine values after cisplatin administration in rats may be reduced by pretreatment with organic acids and bases (Natochin *et al.*, 1987), though there was no reduction of renal platinum accumulation or renal tissue swelling when these were used. The lack of information on the effect of dithiocarbamates on certain aspects of cisplatin nephrotoxicity lead us to investigate the effect of dithiocarbamate treatment on several of these: the ability of the kidney to excrete water and electrolytes, the water and electrolyte content in renal tissue, and platinum accumulation in the kidney. In the present study we examine the effect of dithiocarbamates on cisplatin-induced renal toxicity as measured by these parameters. The dithiocarbamates used were previously reported as inhibitors of cisplatin nephrotoxicity (Jones *et al.*, 1986). These are the hydroxyl-containing dithiocarbamates sodium dihydroxyethyl dithiocarbamate (NaY) and sodium N-methyl-D-glucamine dithiocarbamate (NaG).

### Materials and methods

All experiments were carried out using female Wistar rats weighing between 140–160 g. The animals were injected intraperitoneally with cisplatin (Bristol Laboratories, Syracuse, New York) at a dose of 5 mg $kg^{-1}$ body weight. A preliminary study showed that intravenous or intraperitoneal administration of the cisplatin followed by dithiocarbamate administration resulted in equivalent protection against nephrotoxicity and equal decreases in renal platinum levels. For those animals also given a dithiocarbamate, the dithiocarbamate was administered at a level of 1.71 mmol/$kg^{-1}$ i.p. at 1 and 3 h post cisplatin, i.e., 350 mg NaY $kg^{-1}$ or 500 mg NaG $kg^{-1}$ at each time. NaY and NaG were prepared as described previously (Shinobu *et al.*, 1983; Shinobu *et al.*, 1984). On the fifth day following cisplatin administration a water load of 5 ml per 100 g of body weight was instilled into the stomach via a gastric tube and urine was then collected for the next 2 h. Animals were subsequently decapitated under light ether anesthesia and the kidneys were immediately removed. The water content of the renal tissue was determined by drying at 105°C and electrolyte composition of dry solids was measured after ashing in concentrated nitric acid. The concentration of sodium and potassium in serum, urine and tissue samples was measured with a Flapho-4 flame photometer (Zeiss) and calcium and magnesium were determined by means of a Hitachi 508 atomic absorption spectrophotometer. The urea concentration was measured by reaction with diacetyl monoxime, the creatinine by reaction with picric acid, and the platinum content by neutron activation analysis (Zedgenidze *et al.*, 1980). Statistical evaluations of data were made by means of the Student's *t*-test.

Correspondence: M.M. Jones, Box 1583, Station B, Vanderbilt University, Nashville, Tennessee 37235, USA.
Received 24 January 1990; and in revised form 25 September 1990.

### Results

The glomerular filtration rate (GFR), estimated from creatinine clearance ($C_{Cr}$) values in Table I, markedly decreased on the fifth day following cisplatin administration, dropping to 40% of the control value (Table I). The administration of NaG fully prevented the reduction of the GFR, while NaY was less effective in this respect.

In animals treated with cisplatin and NaG and subsequently given a water load, the water excretion (V) was practically equal to that in control rats (Table I). However, the combination of cisplatin with NaY did not prevent a reduction of water load excretion nor a decrease of the creatinine clearance following cisplatin administration (Table I). Cisplatin treatment in the rat resulted in a decrease in GFR and a rise in both serum creatinine and urea levels; these were obviated by NaG treatment, (Table I).

The regulation of ionic balance by the kidney after cisplatin treatment was relatively effective in maintaining ionic homeostasis on the whole, though there were a moderate increase in the serum magnesium level and a decrease in the serum calcium level. Treatment with NaG eliminated these disturbances in serum composition (Table II). There were a few significant differences among the groups in the excretion of electrolytes by the kidney during the 2 h following the administration of the water load (Table II); the excretion of calcium is modestly elevated in the animals receiving cisplatin.

**Table I** Diuresis (V), serum creatinine ($P_{Cr}$), serum urea ($P_{Ur}$), urinary creatinine ($U_{Cr}$) and urinary urea ($U_{Ur}$) in rats on the fifth day after cisplatin (5 mg $kg^{-1}$ i.p.) administration

| *Group* | *n* | *V* *ml* $hr^{-1}$ *100* $g^{-1}$ *body weight* | $P_{Cr}$ *mg* $dl^{-1}$ | $U_{Cr}$ *mg* $dl^{-1}$ | $U_{Cr}/P_{Cr}$ | $C_{Cr}$ *ml* $h^{-1}$ *100* $g^{-1}$ *body weight* | $P_{Ur}$ *mg* $dl^{-1}$ | $U_{Ur}$ *mg* $dl^{-1}$ |
|---|---|---|---|---|---|---|---|---|
| Control | 10 | 1.62±1.19 | 0.78±0.19 | 15.5±1.3 | 20.6±6.6 | 33.3±9.5 | 37.1±4.4 | 628±193 |
| Cisplatin | 8 | 1.38±0.45 | 1.95±0.79[a] | 15.8±4.2 | 9.2±12.8[a] | 13.6±7.9[a] | 146±96.5[a] | 651±286 |
| Cisplatin + NaY | 8 | 1.15±0.68 | 1.32±0.82 | 19.2±7.6 | 15.5±4.5 | 17.5±17.0[b] | 80.0±82.1 | 807±456 |
| Cisplatin + NaG | 8 | 1.67±0.40 | 0.79±0.25 | 14.2±1.1 | 19.1±5.1 | 32.0±12.2 | 43.6±10.5 | 515±113 |

Results are expressed as mean values±s.d. [a]Significantly different from control values, $P \leqslant 0.01$. [b]Significantly different from control values, $P \leqslant 0.05$.

**Table II** Concentrations in serum (P, mmol $l^{-1}$) and excretion of electrolytes by the kidney (U·V, μmol $h^{-1}$ 100 $g^{-1}$ body weight) following a water load on the fifth day after cisplatin administration (5 mg $kg^{-1}$, i.p.)

| *Group* | *n* | $P_{Na}$ *mmol/l*$^{-1}$ | $U_{Na}\cdot V$ *μmol* $h^{-1}$ *100* $g^{-1}$ *wt* | $P_K$ *mmol* $l^{-1}$ | $U_K\cdot V$ *μmol* $h^{-1}$ *100* $g^{-1}$ *wt* | $P_{Ca}$ *mmol* $l^{-1}$ | $U_{Ca}\cdot V$ *μmol* $h^{-1}$ *100* $g^{-1}$ *wt* | $P_{Mg}$ *mmol* $l^{-1}$ | $U_{Mg}\cdot V$ *μmol* $h^{-1}$ *100* $g^{-1}$ *wt* |
|---|---|---|---|---|---|---|---|---|---|
| Control | 10 | 139±8.9 | 41.0±41.4 | 4.7±0.79 | 12.7±8.66 | 3.10±0.51 | 0.20±0.09 | 0.78±0.16 | 1.62±0.88 |
| Cisplatin | 8 | 137±4.2 | 21.4±15.7 | 4.2±0.65 | 11.7±2.21 | 2.49±0.45[a] | 0.49±0.31[a] | 1.04±0.14[b] | 1.18±0.57 |
| Cisplatin + NaY | 8 | 137±5.9 | 25.0±25.4 | 4.2±0.96 | 8.38±6.17 | 3.06±1.10 | 0.45±0.23[b] | 0.92±0.23 | 1.52±0.76 |
| Cisplatin + NaG | 8 | 138±2.3 | 24.4±15.7 | 4.5±1.21 | 10.7±4.13 | 2.85±1.02 | 0.46±0.51 | 0.86±0.28 | 1.18±0.31 |

Results are expressed as mean values±s.d. [a]Significantly different from control values, $P \leqslant 0.05$. [b]Significantly different from control values, $P \leqslant 0.01$.

For those animals given cisplatin only, the kidney weight increased by 65% (Table III) by the fifth day following cisplatin administration. The causes of this increase in kidney weight included both swelling and an increase in dry solids. This latter may be due to increased blood content in renal tissue. The use of NaG prevented these changes; the use of NaY did not (Table III).

The swelling of the kidneys was accompanied by an increase in the sodium and calcium contents of renal cortex (Table III). These changes of electrolyte composition of the cortex were largely prevented by the administration of NaG. The protective action of NaY was very slight.

The administration of cisplatin resulted in less pronounced alterations in the electrolyte composition of the outer medulla (Table III). The sodium content was not increased. The potassium and magnesium contents in the outer medulla were the same in the NaG treated animals as in the control. NaY treatment did not result in the maintenance of magnesium levels in the outer medulla.

The platinum content of the renal tissue in the unprotected animals was found to be almost three-fold greater than that in animals treated with dithiocarbamates (Table IV). For the cisplatin-only treated animals the renal platinum content was found to be 29.1 ± 1.1 ppm/dry weight on the fifth day following platinum administration. The administration of either NaG and NaY resulted in lower platinum levels with a reduction to about 40% of the levels in animals given only cisplatin. No significant difference was found between the renal platinum levels obtained with these two compounds, though the extent of the protection furnished by these two compounds was significantly different with respect to the creatinine clearance (Table I) and kidney weights (Table III).

### Discussion

The results obtained indicate that NaG is superior to NaY in preventing the nephrotoxic effects found 5 days after cisplatin is given to rats i.p. at a dose of 5 mg cisplatin $kg^{-1}$ body weight. The postulated mechanism of dithiocarbamate action is via competitive chelation and removal of platinum coordinated to protein-bound -SH groups of the kidney tubule cells (Borch & Pleasants, 1979; Borch *et al.*, 1980). The

**Table III** Kidney weights and water and electrolyte contents in renal tissue on the fifth day after cisplatin administration (5 mg $kg^{-1}$, i.p.)

| *Group* | *n* | *Kidney wt (mg 100* $g^{-1}$ *body wt)* *Wet* | *Dry* |
|---|---|---|---|
| Control | 10 | 800±98 | 182.5±20.5 |
| Cisplatin | 8 | 1320±306[b] | 244.5±26.0[b] |
| Cisplatin + NaY | 8 | 1125±125[b] | 228.1±21.5[b] |
| Cisplatin + NaG | 8 | 860±96 | 186.7±18.1 |

*Cortex values (ions in μmol* $g^{-1}$ *wet weight):*

| *Group* | *n* | *Na* | *K* | *Ca* | *Mg* | $H_2O$ *(g/g dry wt)* |
|---|---|---|---|---|---|---|
| Control | 10 | 55.8±5.47 | 81.9±6.35 | 1.66±0.41 | 7.66±0.47 | 2.95±0.25 |
| Cisplatin | 8 | 64.8±7.47[a] | 72.8±7.44[b] | 3.49±1.50[b] | 7.47±0.62 | 4.10±0.79[b] |
| Cisplatin + NaY | 8 | 67.5±6.28[b] | 78.4±3.42 | 2.54±1.33 | 7.11±0.59 | 3.42±0.28[b] |
| Cisplatin + NaG | 8 | 61.5±4.67[a] | 79.9±5.09 | 1.80±0.20 | 7.80±0.37 | 3.03±0.34 |

*Outer medulla (ion values in μmol/g*$^{-1}$ *wet weight)*

| *Group* | *n* | *Na* | *K* | *Ca* | *Mg* | $H_2O$ *(g/g dry wt)* |
|---|---|---|---|---|---|---|
| Control | 10 | 61.9±6.57 | 79.4±3.03 | 2.28±1.23 | 7.60±0.25 | 4.16±0.73 |
| Cisplatin | 8 | 58.2±10.3 | 76.6±8.10 | 4.09±2.66 | 6.58±0.68[b] | 5.50±0.76[a] |
| Cisplatin + NaY | 8 | 56.3±5.69 | 80.3±4.02 | 2.88±2.04 | 7.04±0.54[a] | 4.33±0.96 |
| Cisplatin + NaG | 9 | 58.7±4.75 | 77.4±5.49 | 1.75±0.37 | 7.65±0.40 | 4.31±0.28 |

Results are expressed as means±s.d.; [a]Significantly different from control, $P \leqslant 0.05$; [b]Significantly different from control, $P \leqslant 0.01$.

**Table IV** Platinum content in renal tissue on the fifth day after cisplatin administration[a]

| *Group* | *Platinum content ($\mu g\ g^{-1}$, dry weight)* |
|---|---|
| Control | 0 |
| Cisplatin | 29.1±3.5 |
| Cisplatin + NaY | 11.3±4.0[b] |
| Cisplatin + NaG | 10.9±1.7[b] |

[a]Each animal in the cisplatin groups was given 5 mg cisplatin $kg^{-1}$ i.p. Those animals in the treated groups received either NaY (350 mg $kg^{-1}$) or NaG (500 mg $kg^{-1}$) at 1 and 3 h post cisplatin. Five days later the animals were dissected and tissues removed for analysis. The results are expressed as mean±s.d. [b]Significantly different from cisplatin, $P<0.01$.

distribution of platinum in the kidney following cisplatin administration indicates its specific localisation in the S3 segment of the proximal nephron (Safirstein *et al.*, 1987) and the removal of the platinum from its principal site in producing cellular toxicity should alleviate the renal toxicity, provided this is done soon enough after the administration of the cisplatin. The results obtained show that the administration of dithiocarbamates was followed by a substantial reduction of renal platinum levels (Table IV). However, the results also demonstrate equal renal platinum levels for rats treated with NaY and NaG, though the degree of renal protection achieved by administration of NaG was much greater. Similar effects were observed in earlier studies on cisplatin nephrotoxicity in rats with preliminary administration of organic acids and bases: the renal protection provided by these latter materials was not accompanied by a reduced platinum accumulation in renal tissue (Natochin *et al.*, 1987). The lack of a correlation between renal platinum levels and the degree of renal damage in animals treated to reduce the nephrotoxic action of the cisplatin has been found for choline chloride, para-aminohippurate and ethacrynic acid (Natochin *et al.*, 1989) as well as L-methionine (Basinger *et al.*, 1990). A similar lack of correlation in other tissues has been found in experiments in which circadian rhythms were utilised to select optimum times for the administration of carboplatin (Boughattas *et al.*, 1988).

A large portion of the total platinum that accumulates in kidney cells following cisplatin administration is a product of the biotransformation of cisplatin and these products are not mutagenic (Safirstein *et al.*, 1984). As the mutagenic activity of platinum coordination complexes is correlated with their cellular toxicity (Lecointe *et al.*, 1979), the loss of mutagenicity suggests that such products are less toxic.

It is possible that the differences in the reduction of nephrotoxicity for the two compounds examined here, in spite of the equal reductions of renal platinum levels, involves some specific interaction of the dithiocarbamates with cellular macromolecules which results in a reduced cisplatin binding or in an enhanced level of a non-toxic platinum compound. The results obtained here are of interest in showing that renal electrolyte homeostasis may not be perfectly preserved subsequent to cisplatin administration even in cases where compounds such as dithiocarbamates are administered to protect renal function. While these compounds are capable of reducing renal platinum levels and of maintaining serum levels of creatinine and urea, one may not assume from such data that renal control of electrolyte homeostasis is unimpaired. Most previous studies of the use of various compounds for the protection of renal function against the action of cisplatin have concentrated on the serum non-electrolytes, creatinine and urea, and paid scant attention to electrolyte homeostasis. The present results suggest that this oversight might be unjustified, particularly as the dosage of cisplatin is increased.

### References

BASINGER, M.A., JONES, M.M. & HOLSCHER, M.A. (1990). L-Methionine suppresses pathological sequelae of cis-platinum in the rat. *Fund Appl. Toxicol.*, **14**, 568.

BORCH, R.F. & PLEASANTS, M.E. (1979). Inhibition of cis-platinum nephrotoxicity by diethyldithiocarbamate rescue in a rat model. *Proc. Natl Acad. Sci. USA*, **76**, 6611.

BORCH, R.F., KATZ, J.C., LIEDER, P.H. & PLEASANTS, M.E. (1980). Effect of diethyldithiocarbamate rescue on tumor response to cis-platinum in a rat model. *Proc. Natl Acad. Sci. USA*, **77**, 5441.

BOUGHATTAS, N.A., LÉVI, F., HECQUET, B. & 4 others (1988). Circadian time dependence of murine tolerance for carboplatin. *Toxicol. Appl. Pharmacol.*, **96**, 233.

JONES, M.M., BASINGER, M.A., MITCHELL, W.M. & BRADLEY, C.A. (1986). Inhibition of cis-diamminedichloroplatinum(II)-induced renal toxicity in the rat. *Cancer Chemother. Pharmacol.*, **17**, 38.

LECOINTE, P., MACQUET, J.-P. & BUTOUR, J.-L. (1979). Correlation between the toxicity of platinum drugs to L1210 leukemia cells and their mutagenic properties. *Biochem. Biophys. Res. Comm.*, **90**, 209.

NATOCHIN, YU.V., MYAZINA, E.M., REZNIK, L.V., BROVTSYN, V.K., BAKHTEEVA, V.T. & IVANOV, V.B. (1987). Use of organic acids and bases for the prevention of renal function disorders after cisplatin injection. *Pathol. Physiol. Exp. Ther. (Russ)*, **N2**, 65.

NATOCHIN, YU.V., REZNIK, L.V., BAKHTEEVA, E.M. & BROVTSYN, V.K. (1989). Cisplatin: nephrotoxic action in vertebrates and its prevention. *Comp. Biochem. Physiol.*, **94C**, 115.

QAZI, R., CHANG, A.Y.C., BORCH, R.F. & 4 others (1988). Phase I clinical and pharmacokinetic study of diethyldithiocarbamate as a chemoprotector from toxic effects of cisplatin. *J. Natl Cancer Inst.*, **80**, 1486.

SAFIRSTEIN, R., MILLER, P. & GUTTENPLAN, J.B. (1984). Uptake and metabolism of cisplatin by rat kidney. *Kidney Int.*, **25**, 753.

SAFIRSTEIN, R., WINSTON, J., MOEL, D., DIKMAN, S. & GUTTENPLAN, J. (1987). Cisplatin nephrotoxicity: insights into mechanism. *In. J. Androl.*, **2**, 325.

SHINOBU, L.A., JONES, S.G. & JONES, M.M. (1983). Mobilization of aged cadminum deposits by dithiocarbamates. *Arch. Toxicol.*, **54**, 235.

SHINOBU, L.A., JONES, S.G. & JONES, M.M. (1984). Sodium N-methyl-D-glucamine dithiocarbamate and cadmium intoxication. *Acta Pharmacol. Toxicol.*, **54**, 189.

ZEDGENIDZE, G.A., BROVTSYN, V.K., ROMANOVA, Z.F., SYZKYN, A.B. & TRESHCHALIN, I.D. (1980). Neutron activation analysis in pharmacokinetics of cis-dichlorodiammineplatinum (experimental studies). *Med. Radiol. (Russ)*, **80**, 3.